# Multicenter phase III randomized trial comparing laparoscopy and laparotomy for colon cancer surgery in patients older than 75 years: the CELL study, a Fédération de Recherche en Chirurgie (FRENCH) trial

**DOI:** 10.1186/s12885-019-6376-8

**Published:** 2019-12-04

**Authors:** Gilles Manceau, Antoine Brouquet, Pascal Chaibi, Guillaume Passot, Olivier Bouché, Murielle Mathonnet, Jean-Marc Regimbeau, Rea Lo Dico, Jérémie H. Lefèvre, Frédérique Peschaud, Olivier Facy, Enrico Volpin, Elie Chouillard, Laura Beyert-Berjot, Marc Verny, Mehdi Karoui, Stéphane Benoist

**Affiliations:** 1 Department of Digestive and Hepato-Pancreato-Biliary Surgery, Sorbonne University, Assistance Publique Hôpitaux de Paris, Pitié-Salpêtrière Hospital, Paris, France; 2 Department of Surgery, Paris-Sud University, Assistance Publique Hôpitaux de Paris, Bicetre Hospital, Le Kremlin-Bicetre, France; 3 Unité d’onco-hémato-gériatrie, Sorbonne University, Assistance Publique Hôpitaux de Paris, Charles Foix Hospital, Ivry-sur-Seine, France; 4 Department of Surgical Oncology, CHU Lyon Sud, Hospices Civils de Lyon, Lyon, France; 5 Department of Digestive Oncology, Reims University Hospital, Reims, France; 6 Department of Digestive and Endocrine Surgery, Dupuytren University Hospital, Limoges University, Limoges, France; 7 Department of Digestive and Oncological Surgery, Amiens University Hospital, Amiens, France; 8 Department of Visceral and Oncologic Surgery, Paris Diderot University, Assistance Publique - Hôpitaux de Paris, Saint-Louis Hospital, Paris, France; 9 Department of Surgery, Sorbonne University, Assistance Publique - Hôpitaux de Paris, Saint-Antoine Hospital, Paris, France; 10 Department of Digestive, Oncologic and Metabolic Surgery, Versailles St-Quentin-en-Yvelines/Paris Saclay University, Assistance Publique - Hôpitaux de Paris Ambroise Paré Hospital, Boulogne-Billancourt, France; 11 Department of Digestive Surgical Oncology, Dijon University Hospital, Dijon, France; 12 Department of visceral and urological surgery, Simone Veil Hospital, Eaubonne, France; 13 Department of Minimally Invasive Surgery, Poissy Saint Germain Medical Center, Poissy, France; 14 Department of Digestive Surgery, Aix-Marseille Université, Marseille, France; 15 Department of Geriatrics, Sorbonne University, Assistance Publique - Hôpitaux de Paris, Pitié-Salpêtrière Hospital, Paris, France

**Keywords:** Elderly patient, Colon cancer, Surgery, Laparoscopy, Laparotomy, Morbidity, Phase III trial

## Abstract

**Background:**

Several multicenter randomized controlled trials comparing laparoscopy and conventional open surgery for colon cancer have demonstrated that laparoscopic approach achieved the same oncological results while improving significantly early postoperative outcomes. These trials included few elderly patients, with a median age not exceeding 71 years. However, colon cancer is a disease of the elderly. More than 65% of patients operated on for colon cancer belong to this age group, and this proportion may become more pronounced in the coming years. In current practice, laparoscopy is underused in this population.

**Methods:**

The CELL (Colectomy for cancer in the Elderly by Laparoscopy or Laparotomy) trial is a multicenter, open-label randomized, 2-arm phase III superiority trial. Patients aged 75 years or older with uncomplicated colonic adenocarcinoma or endoscopically unresectable colonic polyp will be randomized to either colectomy by laparoscopy or laparotomy. The primary endpoint of the study is overall postoperative morbidity, defined as any complication classification occurring up to 30 days after surgery. The secondary endpoints are: 30-day and 90-day postoperative mortality, 30-day readmission rate, quality of surgical resection, health-related quality of life and evolution of geriatric assessment. A 35 to 20% overall postoperative morbidity rate reduction is expected for patients operated on by laparoscopy compared with those who underwent surgery by laparotomy. With a two-sided α risk of 5% and a power of 80% (β = 0.20), 276 patients will be required in total.

**Discussion:**

To date, no dedicated randomized controlled trial has been conducted to evaluate morbidity after colon cancer surgery by laparoscopy or laparotomy in the elderly and the benefits of laparoscopy is still debated in this context. Thus, a prospective multicenter randomized trial evaluating postoperative outcomes specifically in elderly patients operated on for colon cancer by laparoscopy or laparotomy with curative intent is warranted. If significant, such a study might change the current surgical practices and allow a significant improvement in the surgical management of this population, which will be the vast majority of patients treated for colon cancer in the coming years.

**Trial registration:**

ClinicalTrials.gov NCT03033719 (January 27, 2017).

## Background

Colorectal cancer (CRC) is the third site of cancer worldwide with over 1.2 million new diagnosed cases annually [1]. Colon cancer is a disease of the elderly. Patients older than 85 years are three times more likely to develop colon tumors than those aged between 60 and 69 years [2]. The proportion of elderly patients managed for colon cancer can only increase in the future, due to the increase in life expectancy and the aging of the general population [3, 4].

Several randomized trials have demonstrated that colon cancer surgery should be performed by laparoscopy [5, 6]. Compared to open surgery, laparoscopic approach significantly improves postoperative recovery and short-term outcomes [7–15], with no adverse effect on long-term oncological outcomes [16–21]. However, patients recruited in these phase III trials were significantly younger that patients operated on in daily practice, with a median age not exceeding 71 years [7–15]. We can hypothesize that benefit of mini-invasive surgery is even more pronounced in frailer, older colon cancer patients because of improved postoperative comfort, lesser postoperative analgesics consumption and earlier recovery compared to open usrgery. However, laparoscopy has been consistently associated with longer operative duration and, whether this negatively affects outcome by increasing geriatric complications including postoperative delirium in the elderly is unknown. When we look at surgical practices, we see that the age of patients has an influence on the approach used. In a recent French survey of more than 84,000 patients who underwent colorectal resection for cancer between 2006 and 2008 and based on data from the national prospective database French Medical Information System (PMSI, *Programme de Médicalisation des Systèmes d’Information*), Panis and colleagues [22] reported that patients older than 70 years were significantly less operated by laparoscopy compared with younger patients (21.7% vs. 32.9%, *p* < 1.10–6). Therefore, as elderly patients have been excluded from the major studies that led to the development of guidelines for the surgical management of colon cancer, whether these recommendations can be extrapolated to the elderly population or not is unknown.

## Methods/design

### Protocol overview

**Fig. 1:**
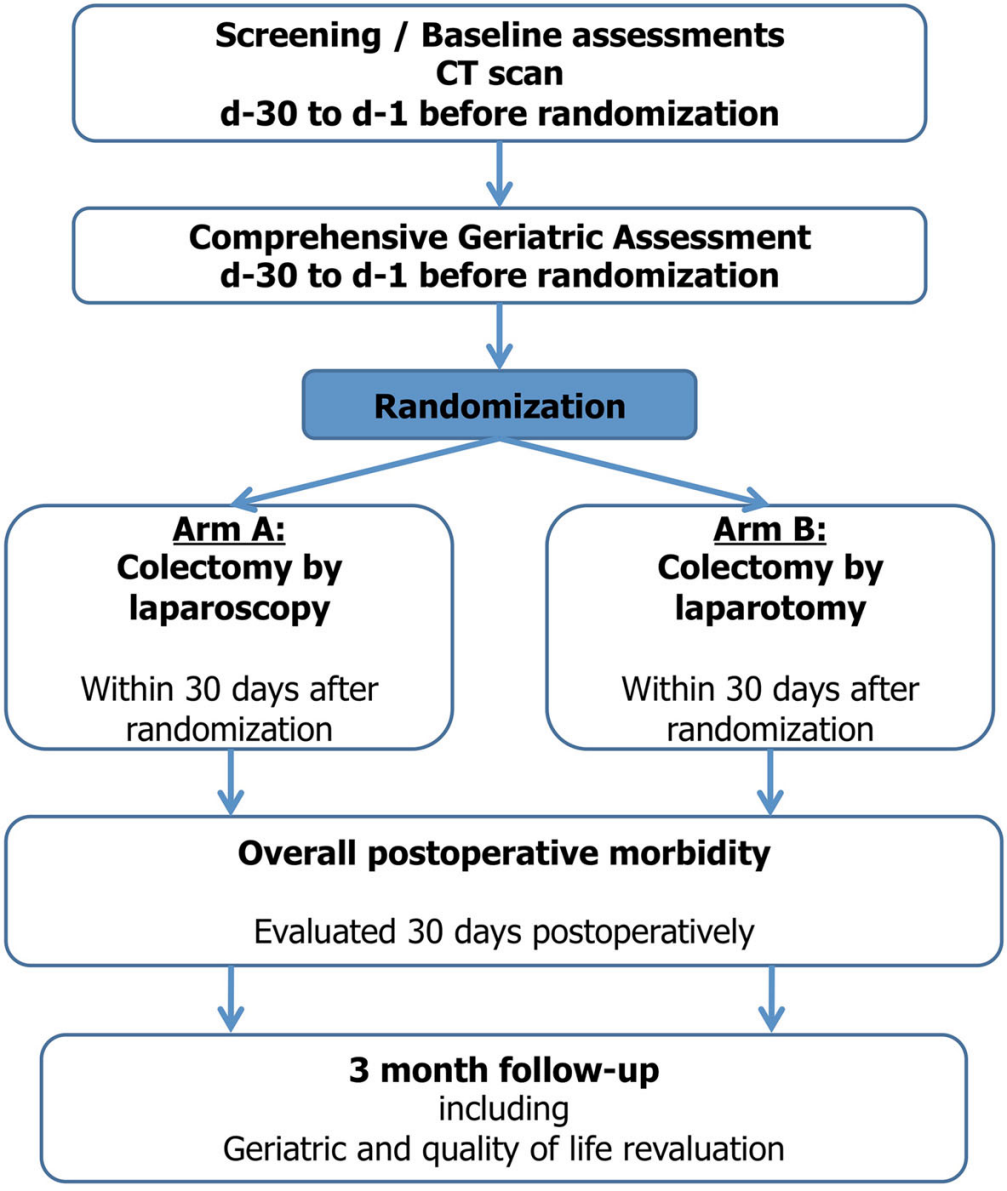
Temporal sequence of trial conduct in patients included in this trial

The CELL (Colectomy for cancer in the Elderly by Laparoscopy or Laparotomy) trial is a multicenter, open-label randomized, 2-arm phase III superiority trial comparing laparoscopic or open colectomy in elderly patients operated on for colon cancer. For all patients likely to be included in this trial, a comprehensive geriatric assessment (CGA) must be performed within 30 days before the randomization by a geriatrician of the investigator center. This multidimensional evaluation is a major consideration in assessment of operative risk, treatment decision-making, and adapting perioperative care if surgery is undertaken. It aims to determine the medical, psychological and functional capabilities of elderly persons and to distinguish the weakest patients. This diagnostic process will include several reproducible and internationally validated tools, with at least the following scores: the Mini Mental State (MMS) test, the Katz Activities of Daily Living (ADL) scale, the Lawton Instrumental Activites of Daily Living (IADL) scale, the Geriatric Depression Scale (GDS), and the Timed Get-up-and-go (TGUG) test. The general treatment plan is given in Fig. 1.

### Inclusion criteria

For inclusion in the study, all of the following inclusion criteria must be fulfilled: (i) age ≥ 75 years; (ii) histologically proven colonic adenocarcinoma (> 15 cm from the anal margin) or endoscopically unresectable colonic polyp; (iii) uncomplicated primary tumor (preoperative suspicion of invasion of adjacent structures by the colon cancer (cT4) on CT scan, tumor perforation, obstruction, abscess, bleeding); (iv) no history of colorectal cancer in the last 5 years; (v) no peritoneal carcinomatosis on abdominal CT scan; (vi) patient able to fill in an auto-questionnaire alone or with some help; (vii) patient who has signed an informed consent form prior to randomization and who commits himself or herself to respect the protocol instructions; (viii) positive CGA and MMS (Mini Mental Score) ≥ 15.

### Exclusion criteria

Patients are not eligible for this study if any of the following exclusion criteria apply: (i) rectal cancer (≤ 15 cm from the anal verge); (ii) locally advanced (cT4 tumor) or complicated primary tumor requiring extended local resection or emergency surgery; (iii) synchronous colorectal cancer; (iv) scheduled need for synchronous intra-abdominal surgery, including surgery for liver metastases; (v) absolute contraindications to general anesthesia or prolonged pneumoperitoneum; (vi) patient not able to tolerate colon surgery according to the CGA; (vii) estimated life expectancy less than 6 months; (viii) patient under guardianship; (ix) other known active cancer (except nonmelanomatous skin cancer); (x) patient not affiliated to the social security system.

### Endpoints

The main endpoint of this phase III study is overall postoperative morbidity, defined as any surgical or medical complications occurring up to 30 days after the surgery. If a patient is discharged from the hospital before this delay, an appointment for an outpatient visit will be given at the 30th postoperative day to finish completing the postoperative morbidity form. Postoperative morbidity will be collected by an independent observer, using a standardized collection form during hospitalization and eventual subsequent out-patient visits. The following specific complications will be documented:

- Geriatric complications: postoperative delirium, need for physical restraint, fecal impaction, bedsore, fall, swallowing disorder.
- Postoperative transfusion.
- Abdominal infectious complications: anastomotic leakage, intra-abdominal collection at a distance from the anastomosis, wall abscess or cellulitis.
- Non-infectious abdominal complications: paralytic ileus, hematoma, hemoperitoneum, gastrointestinal bleeding, evisceration.
- Medical complications: cardiovascular complications including cardiac arrest, acute coronary syndrome without myocardial infarction, myocardial infarction, acute heart failure, heart rhythm disorder, transient ischaemic attack or stroke or other; pulmonary complications including pneumonia, pulmonary embolism, acute respiratory failure, pleural effusion or other; deep vein thrombosis; urinary and nephrological complications including acute urinary retention, lower urinary tract infection, pyelonephritis, acute renal failure or other; catheter complications including lymphangitis, thrombosis, infection or other.

The occurrence of postoperative delirium is a specific complication reported in elderly patients after surgery [23]. This complication will be specifically assessed using the Confusion Assessment Method, an instrument validated for the diagnosis of delirium [24]. Anastomotic leakage will be defined either with the clinical findings of peritonitis or radiologically by computed tomography scan with water-soluble rectal contrast demonstrating endoluminal extravasation or isolated intra-abdominal abscess close to the anastomosis. It will be recorded even if it did not involve surgical management. Post-operative complications will be graded from 0 to V based on the classification system validated by Clavien and Dindo [25].

Secondary endpoints are the following: (i) 30-day and 90-day postoperative mortality; (ii) readmission rate. A readmission will be defined in both arms as any rehospitalization within 30 days after discharge from the hospital; (iii) quality of surgical resection. Surgical resection, performed either by laparoscopy or laparotomy, must achieve oncological quality criteria recommended by the French Federation of Digestive Oncology (FFCD, *Fédération Française de Cancérologie Digestive*) and by the French National Authority for Health (HAS, *Haute Autorité de Santé*). Quality and radicality of the surgical excision will be evaluated on histological criteria (number of lymph nodes harvested, R0 resection and mesocolic resection quality in 3 grades); (iv) health-related quality of life. Quality of life will be assessed using the French version the European Organization for Research and Treatment of Cancer Quality of Life Questionnaire Core 30 (EORTC QLQ-C30) version 3.0, with the specific CRC module (QLQ-CR29) [26, 27]. Quality of life will be assessed at the inclusion before surgery and 3 months postoperatively in both treatment arms. The QLQ-C30 consists of 30 items with both multi-items scales and single item measures. The internal validation of QLQ-C30 allowed to identify 15 scales and to generate 15 scores: 5 scores of functional scale, a global health status scale and 9 symptoms scale. All EORTC QLQ-C30 raw scores will be linearly transformed to a value ranging from 0 to 100 according to the EORTC scoring manual. For the 5 functional scales (physical, role, cognitive, emotional and social) and global quality of life scale, a higher score represents a higher level of functioning or quality of life. For the 9 symptom scales and items (fatigue, nausea and vomiting, pain, dyspnea, sleep disturbance, appetite loss, constipation, diarrhea and financial difficulties), a higher score corresponds to a higher degree of symptoms; (v) evolution of geriatric scores (MMS, ADL scale, IADL scale, GDS and TGUG test) at 3 months postoperatively in both arms.

All data of interest will be entered electronically on the Cleanweb site and stored in the patient’s file. An auditing trial will be performed by the Clinical Research Unit of Pitié-Salpêtrière hospital at least twice in each participating center.

### Randomization

After completion of all the screening evaluations and the CGA, a second visit will be performed. All eligible patients will be randomly assigned to one of the treatment arms after ensuring that all the inclusion criteria are satisfied, none of the exclusion criteria apply, and the patient gave his nonopposition to participate in the study. The randomization will be performed electronically on the Cleanweb site (https://cleanweb.aphp.fr/Ctms-02/portal/login). To obtain homogeneous populations among treatment arms, patients will be randomized by minimization (in a 1:1 ratio) owing to the following stratification factors: (i) investigator center; (ii) colon tumour location (right or left side, with respect to the splenic flexure); (iii) stage IV colon cancer (excluding peritoneal disease); (iv) age (75–85 years vs. >85 years).

### Intraoperative and postoperative management

Neither patients nor health-care providers will be blinded to patient groupings.

#### Arm A: colectomy by laparoscopy

The colectomy will be performed within 30 days after the randomization, by laparoscopy. The extraction site of the specimen is left to the investigator’s discretion. For left colectomy, colorectal anastomosis must be performed laparoscopically with stapled colorectal anastomosis. The type of anastomosis (stapled or hand-sewn) during right colectomy is left to the surgeon’s discretion.

The decision to convert to conventional surgery will be made by the surgical team. Conversion is defined as an inability to complete all intended laparoscopic steps laparoscopically.

#### Arm B: colectomy by laparotomy

The colectomy will be performed within 30 days after the randomization, by laparotomy. For right colectomy, surgery can be performed by midline or right transverse laparotomy. The type of anastomosis (stapled or hand-sewn) either for right or left colectomy is left to the surgeon’s discretion.

#### Common management in both arms

Immuno-nutrition will be given 7 days prior colectomy following the 2011 recommendation from High Authority for Health (HAS) and French clinical guidelines from the French Society of Anaesthesiology and Reanimation (SFAR, Société Française d’Anesthésie et de Réanimation) [28]. No mechanical bowel preparation will be administered before surgery [29]. Anesthetic evaluation will be performed according to the local practices of each investigator center. Epidural analgesia can be performed but is not required in the present trial. Antibiotic prophylaxis and thrombosis prophylaxis will be done according to local standards without consideration of group designation. Whatever the surgical approach, colectomy will be performed with respect to the oncologic labelled HAS-INCa standards recommendations (determined by the FFCD and updated in 2009 by the Evaluation Commission of the SFCD, *Société Française de Chirurgie Digestive*) [30]. According to these recommendations, the colectomy should be performed with curative intent. Neither prophylactic abdominal drainage nor nasogastric tube should be left in place at the end of the procedure [29, 31]. In the postoperative period, enhanced recovery principles are recommended in both arms but no specific perioperative geriatric management protocol is used in the context of the research. Semi-liquid diet should be given on the first postoperative day. Apart from history of prostatic disease, the bladder catheter should be removed on the first postoperative day. The use of tramadol is not recommended because of the reported increased confusional risk [23].

### Statistical analysis and sample size

This study is multicenter, open-label randomized, 2-arm phase III superiority trial, comparing arm A (colectomy by laparoscopy) versus arm B (colectomy by laparotomy) for patients aged 75 years old or more with uncomplicated colon cancer. The hypotheses for sample size calculation are: (i) H0: there is no difference in overall postoperative morbidity between the two arms; (ii) H1: there is a significant difference, with estimated global morbidities in the laparoscopy and laparotomy groups of 20 and 35%, respectively. With a two-sided α risk of 5% and a power of 80% (β = 0.20) as alternative hypothesis H1, 276 patients will be included in total (138 patients in each arm). No interim analysis will be performed. The endpoints will be analyzed according to the intention-to-treat principle, in such a way that patients who did not receive their allocated surgical procedure were analyzed in the treatment group to which they had been randomized. An additional as-treated analysis will also be done, taking into account intraoperative conversions to the open-surgery group.

Continuous variables will be described via the mean, standard deviation, median, minimum and maximum. Categorical variables will be described using frequencies and percentages. The percentages will be calculated with the missing data item. 95% confidence intervals (95% CI) will be calculated when necessary.

Percentage differences between groups will be compared with the Pearson’s χ^2^ test or Fisher’s exact test, as appropriate. Comparison of continuous data will be done by use of the Student’s t test or the nonparametric Mann-Whitney U test, depending on their distribution. Two-tailed *P*-values <0.05 will be considered statistically significant.

Quality of life will be described preoperatively and 3 months postoperatively in both treatment arms. Rate of patients having a QLQ-C30 or QLQ-CR29 score improved, deteriorated or stabilized at the second quality of life assessment will be reported in each arm with frequency and percent. The weighted means at baseline (preoperative score) and at 3 months will be compared in each arm using the Wilcoxon’s signed-rank test. Based on the study by Osoba and colleagues [32], this analysis will be limited to domains showing a difference of at least 10 points between the two assessments, which can be interpreted as a clinically important change. Mean quality of life changes from baseline will be computed in each arm with 95% CI. The weighted mean differences between both arms will be compared using the nonparametric Mann-Whitney U test.

### Participating centers

Seventeen French centers will participate in this study: Beaujon University Hospital in Clichy, Claude Huriez University Hospital in Lille, Dupuytren University Hospital in Limoges, Intercommunal Hospital Center of Poissy-Saint-Germain-en-Laye, Le Kremlin-Bicêtre University Hospital, Pitié-Salpêtrière University Hospital in Paris, Saint-Antoine University Hospital in Paris, University Hospital of Amiens, University Hospital of Rouen, North University Hospital in Marseille, University Hospital of Reims, Pierre Benite University Hospital in Lyon, Simone Veil Hospital in Eaubonne, Ambroise Paré University Hospital in Boulogne, University Hospital of Dijon, Edouard Herriot University Hospital in Lyon, Foch Hospital in Suresnes.

### Ethics and safety

This study protocol was approved by the Institutional Review Board of the Ile de France IV ethic committee on July 2016 (ref 2016/26SC). The institutional promoter is the Assistance Publique Hôpitaux de Paris, France. The trial has been registered on ClinicalTrials.gov website under the identification number NCT03033719 on January 2017. This study received a grant from the Ministry of Social Affairs and Health of France. The study complies with the Declaration of Helsinki rules and the principles of the Good Clinical Practices guidelines.

## Discussion

### Specific issues of colon cancer in elderly patients

Elderly people are a heterogeneous group of patients, ranging from very fit to very frail individuals. They raise specific issues, whether at the time of diagnosis of the primary tumor or during surgery and hospitalization. First, this subset of patients has less physiological reserves, less stress tolerance and significantly more comorbidities, notably cardiovascular, pulmonary and neurological diseases [33]. Approximately 70–80% of patients older than 80 years have at least one comorbidity and 75% of them have an ASA score ≥ 3. The prevalence of undernutrition is very important and only 40% of elderly patients have a serum albumin >35 g/L [34]. Secondly, the disease is generally diagnosed at a more advanced stage. They need more emergency surgery and the curative surgery rate is substantially lower [35]. Thirdly, when curative surgery is performed, postoperative complications have more serious consequences. This fragile condition causes excess postoperative morbidity and mortality. A systematic review published in 2000 that included 28 independent studies with a total of 34,194 patients found that the incidence of postoperative mortality increased progressively with advancing age [36]. The mortality rate in the 65–74 year age group was about 1.8 times that of those aged less than 65 years. It rose to about 3.2 in the 75–84 year age group and 6.2 in the 85+ age group. Similarly, postoperative morbidity was significantly higher in elderly patients, notably cardiovascular and pulmonary complications.

Our study, if positive, may change the current surgical practices and bring a significant improvement in the surgical management of this population, which will be the vast majority of patients treated for colon cancer in the coming years.

### Potential benefits of laparoscopic colectomy for cancer in elderly patients

Published studies that reported short-term outcomes after laparoscopic or open colorectal resection in the specific group of elderly patients suggest that laparoscopic technique could be safely used in elderly patients with colorectal cancer (Table 1) [37–43]. This surgical approach is associated with less postoperative morbidity compared with laparotomy, mostly by decreasing medical and cardiopulmonary complications. Global postoperative morbidity rate decreases from 33.2 to 20.3% for patients older than 75 years. These rates were taken into account for the number of patients to include in the present trial.

**Table 1:** Summary table of studies that have reported postoperative results of elderly patients operated on for colon cancer by laparoscopy or laparotomy

Authors	Year	No. of patients	Study population, years	Global postoperative morbidity (%)		Medical morbidity (%)		Cardiopulmonary morbidity (%)	
				Laparoscopy	Laparotomy	Laparoscopy	Laparotomy	Laparoscopy	Laparotomy
Bader [37]	1986	96	> 75	/	39.0	/	17.0	/	9,4
Bardram et al. [38]	2000	50	> 75	16.0	/	2.0	/	0,0	/
Stocchi et al. [39]	2000	42	> 75	14.3	33.3	11.9	26.2	9,5	21,4
Sklow et al. [40]	2003	39	> 75	31.0	31.0	12.9	17.9	7,7	10,3
Latkauskas et al. [41]	2005	116	> 75	/	32.7	/	19.0	/	NR
Hermans et al. [42]	2010	74	> 75	/	43.2	/	51.4	/	14,0
She et al. [43]	2013	434	> 75	20.6	28.6	19.6	24.9	12,7	19,3
Total		851		20.3	33.2	15.0	25.4	9.7	16.0

*NR* Not reported

Two retrospective studies have compared postoperative outcomes of patients operated on for colon cancer by laparoscopy and laparotomy [44, 45]. Allardyce and colleagues [44] performed a retrospective analysis from the Australasian Laparoscopic Colon Cancer Study [9], with a total of 592 patients. While postoperative morbidity was not different between laparoscopy and laparotomy for all included patients and in the group of patients younger than 70 years (45.3% vs. 37.8%, *p* = 0.06 and 30.0% vs. 34.9%, *p* = 0.432, respectively), postoperative morbidity in patients aged 70 years or more was significantly lower in the laparoscopic group (36.8% vs. 50.7%, *p* = 0.019). Similarly, Frasson and colleagues [45] found in a series of 535 patients with colorectal disease that patients aged 70 years or more included in the laparoscopic group had substantially less postoperative complications than those included in the open group (20.2% vs. 37.5%, *p* = 0.01). Kennedy and colleagues [46] used the database of the American College of Surgeons (ACS) National Surgical Quality Improvement Program (NSQIP) to evaluate factors correlated with postoperative morbidity in elderly patients undergoing elective surgery for colon cancer. Between 2005 and 2008, 5914 patients over the age of 65 were studied. The rate of 30-day complications was significantly lower for patients operated on by laparoscopy compared with those operated on by laparotomy (16.1% vs. 25.4%, *p* < 0.005). Open surgery was one of the factors associated with an increased risk of complications in multivariate analysis.

To our knowledge, only one randomized trial compared laparoscopic surgery and conventional open surgery specifically in patients over 75 years of age operated on electively for colorectal cancer [47]. However, this trial was a non-inferiority study, with as primary endpoint 3-year recurrence-free survival. And specific assessment of postoperative complication was not performed. Nevertheless, with 200 patients recruited between 2008 and 2012 in a single institution, the authors showed that the overall postoperative morbidity rate was significantly lower in the laparoscopic group than in the open surgery group (22% vs. 36%, *p* = 0.029).

### Defining “the elderly” and choice of study population

Aging can be broadly defined as a progressive, generalized impairment of function resulting both in a loss of adaptive response to a stress and in a growing risk of age-associated disease [48]. Although ageing is a complex, multifactorial process, patients are often defined as elderly based on their chronological age. In this trial, the threshold age of 75 years is based on the results of the study by Kurian and colleagues [48]. With the use of the ACS-NSQIP database, the authors identified 129,331 patients who underwent major gastrointestinal resections, with notably 101,258 colectomies (78.3% of included patients). In this large study, it was observed an accelerate increase in the 30-day mortality rate starting at 75 years of age (5.3% per decade). Thus, based on the most recent literature, we consider that for patients requiring colon surgery for cancer the chronologic age of 75 years is a relevant cutoff to define “the Elderly”. As defined by WHO, our study population will include the “middle old” (75–85 years) and the “old old” (85 years or more). Our study will also be stratified on these age’s intervals. However, as age alone cannot properly reflect ageing, all patients who will be included in this trial will have a preoperative CGA with the use of validated scores.

### Choice of the primary endpoint and general study design

Many retrospective studies suggest that laparoscopy decreases postoperative morbidity of colectomy for cancer in elderly patients [37–43]. The positive impact of laparoscopy on morbi-mortality should however be interpreted with caution. The methodological quality of these studies was generally poor and the populations studied heterogeneous. Almost all of them were single-centre retrospective comparative studies or case-matched studies, with inherent selection biases. Because no dedicated randomized study has been undertaken to assess the benefits of laparoscopic surgery for colon cancer in terms of postoperative morbidity in elderly patients, we decided to design the present multicenter, randomized, open label phase III superiority trial.

## Data Availability

The datasets used and/or analyzed during the current study are available from the corresponding author on reasonable request.

## Abbreviations

ACS: American College of Surgeons
ADL: Activities of Daily Living
CELL: Colectomy for cancer in the Elderly by Laparoscopy or Laparotomy
CGA: Comprehensive geriatric assessment
CRC: Colorectal cancer
FFCD: Fédération Française de Cancérologie Digestive
GDS: Geriatric Depression Scale
HAS: Haute Autorité de Santé
IADL: Instrumental Activites of Daily Living
INCa: Institut National du Cancer
MMS: Mini Mental State
NSQIP: National Surgical Quality Improvement Program
SFCD: Société Française de Chirurgie Digestive
TGUG: Timed Get-up-and-go
